# Dibutyl phthalate promotes juvenile Sertoli cell proliferation by decreasing the levels of the E3 ubiquitin ligase Pellino 2

**DOI:** 10.1186/s12940-020-00639-1

**Published:** 2020-08-01

**Authors:** Tan Ma, Jiwei Hou, Yuan Zhou, Yusheng Chen, Jiayin Qiu, Jiang Wu, Jie Ding, Xiaodong Han, Dongmei Li

**Affiliations:** 1 Immunology and Reproduction Biology Laboratory & State Key Laboratory of Analytical Chemistry for Life Science, Medical School, Nanjing University, Nanjing, 210093 Jiangsu China; 2 Jiangsu Key Laboratory of Molecular Medicine, Nanjing University, Nanjing, 210093 Jiangsu China

**Keywords:** Dibutyl phthalate, Monobutyl phthalate, Sertoli cells, Apoptosis, Peli2, Proliferation

## Abstract

**Background:**

A previous study showed that dibutyl phthalate (DBP) exposure disrupted the growth of testicular Sertoli cells (SCs). In the present study, we aimed to investigate the potential mechanism by which DBP promotes juvenile SC proliferation in vivo and in vitro.

**Methods:**

Timed pregnant BALB/c mice were exposed to vehicle, or DBP (50, 250, and 500 mg/kg/day) from 12.5 days of gestation until delivery. In vitro, CCK-8 and EdU incorporation assays were performed to determine the effect of monobutyl phthalate (MBP), the active metabolite of DBP, on the proliferation of TM4 cells, which are a juvenile testicular SC cell line. Western blotting analysis, quantitative PCR (q-PCR), and flow cytometry were performed to analyse the expression of genes and proteins related to the proliferation and apoptosis of TM4 cells. Coimmunoprecipitation was used to determine the relationship between the ubiquitination of interleukin 1 receptor-associated kinase 1 (IRAK1) and the effect of MBP on promoting the proliferation of TM4 cells.

**Results:**

In the 50 mg/kg/day DBP-exposed male mice offspring, the number of SCs was significantly increased. Consistent with the in vivo results, in vitro experiments revealed that 0.1 mM MBP treatment promoted the proliferation of TM4 cells. Furthermore, the data showed that 0.1 mM MBP-mediated downregulation of the E3 ubiquitin ligase Pellino 2 (Peli2) increased ubiquitination of IRAK1 by K63, which activated MAPK/JNK signalling, leading to the proliferation of TM4 cells.

**Conclusions:**

Prenatal exposure to DBP led to abnormal proliferation of SCs in prepubertal mice by affecting ubiquitination of the key proliferation-related protein IRAK1 via downregulation of Peli2.

## Background

Dibutyl phthalate (DBP) is a widely used plasticizer that has a negative effect on the development and function of male reproductive organs in humans and laboratory animals [1, 2]. As DBP binds to the matrix by non-covalent bond, it easily leaches into the environment and then migrates into the food [2]. The toxicological effects of DBP are complex and diverse. Among them, the impact of in utero exposure to DBP on foetal reproduction and development is particularly worthy of concern. Some studies confirmed that in utero exposure to DBP caused testicular malformations in male offspring [3–5], but the underlying mechanism has not yet fully investigated. As one of the target cells of DBP/MBP [5–9], Sertoli cells (SCs) are the first that are recognized to differentiate in the foetal indifferent gonad, and they play a critical role in foetal testis formation and sexual differentiation as well as in adult spermatogenesis [10–12]. Because of the fixed number of germ cells supported by SCs, the proliferative capability of immature SCs during prepuberty determines the number of mature SCs, testis size and output of germ cells in the mature testis. Our recent study suggested that monobutyl phthalate (MBP), the metabolite of DBP, could disrupt the growth of juvenile SCs [9], however, the underlying molecular mechanism still needs to be further explored.

Based on the data generated by screening a high-throughput mRNA microarray, downregulation of E3 ubiquitin ligase Pellino 2 (Peli2) was found in SCs after exposure to 0.1 mM MBP [9]. Peli2, a member of the Pellino protein family, is a novel E3-RING ubiquitin ligase involved in the ubiquitination and degradation of interleukin-1 receptor-related kinase 1 (IRAK1). Previous studies revealed that Peli2 mediated K63-linked IRAK1 polyubiquitination and reduced K48-linked IRAK1 polyubiquitination, thereby leading to the activation of downstream MAPK/JNK signalling pathways [13–15]. The activation of IRAK1 downstream of the MAPK/JNK signalling pathway is related to many cellular processes, such as cell proliferation, migration, and regeneration [16, 17]. Meanwhile, both the extrinsic apoptotic pathway involving the Fas/FasL proteins, such as FADD, and the intrinsic pathway (mitochondria-mediated through the Bax/Bcl-2 family proteins) can regulate cell growth by inducing the apoptosis of SCs [18]. Given these previous studies, we raised the question of whether the Peli2-mediated proliferation pathway as well as apoptotic pathways were involved in MBP-mediated growth disruption of immature SCs.

In this study, we first evaluated the effect of DBP/MBP on proliferation and apoptosis in vivo and in vitro, and then we investigated the molecular mechanism by which MBP promotes the proliferation of TM4 cells.

## Methods

### Animals and processing method

Nine-week-old male (*n* = 12) and female (*n* = 24) specific pathogen-free (SPF) BALB/c mice were obtained from the Experimental Animal Center of the Academy of Military Medical Science, Beijing, China. Time-mated females (day of vaginal plug = gestational day (GD) 0.5) were randomized into 4 groups (*n* = 6 for each group). Pregnant mice were treated with 0 (control), 50, 250, or 500 mg/kg/day DBP (Sigma, St. Louis, USA) in 1 ml/kg corn oil, which was administered daily by oral gavage from GD 12.5 until birth. Because seminiferous cord and gonocyte development of offspring were damaged under the daily oral dose of 500 mg/kg/day DBP given to pregnant mice from GD 16–18 [19], we set 500 mg/kg/day as the highest concentration group. The 22-day-old males were euthanized by CO_2_ asphyxiation. The testes were carefully removed and fixed in 4% paraformaldehyde.

All procedures performed on animals were approved by the Animal Care and Use Committee of Nanjing University under the animal protocol number SYXK (Su) 2009–0017. The animal experiments were performed in accordance with the Guide for the Care and Use of Laboratory Animals (The Ministry of Science and Technology of China, 2006).

### Reagents and cell culture

Foetal bovine serum (FBS), Triton® X-100, DMEM-F12 and MBP were purchased from Sigma-Aldrich Inc. (St. Louis, MO, USA). MBP (2.2224 g) was dissolved in 1 mL of DMSO to prepare a stock solution (10 M). SP600125 (JNK inhibitor) and an IRAK1 inhibitor were purchased from MedChemExpress (Monmouth Junction, NJ, USA). The antibodies used in this study are listed in Additional file 1: Table S1. TM4 cells were cultured in DMEM/F12 containing 10% FBS and 1% penicillin-streptomycin with a 5% CO_2_ atmosphere in a humidified incubator at 37 °C. TM4 cell lines were obtained from the American Type Culture Collection (Manassas, VA, USA).

### Immunohistochemical analyses

Immunohistochemical analyses were carried out as previously described [20]. The primary and secondary antibodies used in this study were SOX9, Peli2, and HRP-conjugated secondary antibodies (Zhongshan Biotechnology, Beijing, China). For each section, ten images were randomly captured at 200× magnification under a light microscope. The total cells and the SOX9- or Peli2-positive cells in each image were counted automatically using ImageJ software. After calculating the average of ten images, excluding the minimum and maximum values, the positive ratio of SOX9- or Peli2-expressing cells was determined; six sections per group of mice were taken for statistical analysis.

### Cell growth assay

A Cell Counting Kit-8 (CCK-8) (Dojindo Lab., Kumamoto, Japan) test was used to test cell growth after treatment with MBP according to the manufacturer’s instructions. Briefly, TM4 cells were plated at 2 × 10^3^ cells per well in 96-well culture plates. After 24 h, cells were treated with MBP at concentrations of 0, 0.1, 1 or 10 mM for various times (1, 2, 3, 4, or 5 days). Based on our previous study of cell viability, the median effective concentration (EC_50_) of MBP was determined to be 16.21 mM [21]. In this study, the highest concentration of MBP used was 10 mM. Following MBP treatment, 100 μL of a mixed solution of 1:10 (v/v) CCK-8:DMEM/F12 was added to each well, and the cells were incubated for an additional 4 h. Absorbance was measured at the indicated time points at 450 nm with a microplate reader (Versamax, Chester, PA, USA). CCK-8 contains WST-8, which can be reduced by dehydrogenases in cells to generate an orange-coloured product (formazan), which is soluble in the tissue culture medium. Therefore, the amount of formazan dye generated by dehydrogenases in cells is directly proportional to the number of living cells. Measurements were performed at least three times on six samples in parallel. Cell survival rate = (As-Ab)/(Ac-Ab) * 100%, and the terms are defined as follows: As: experiment well; Ab: blank well; and Ac: control well.

### EdU incorporation assay

EdU assay kits were used to determine cell proliferation (Click-iT® EdU Imaging Kits; Invitrogen). According to the kit’s instructions, 1 mL of proliferation media containing 20 μM EdU (final concentration 10 μM) was added to 6 wells of the plate, containing cells to be incubated with final concentrations of 0, 0.1, 1 or 10 mM MBP for 24 h. Cells were then fixed with 4% paraformaldehyde for 15 min. The fixative was removed, and the cells were washed twice with 1 mL of 3% bovine serum albumin (BSA), which was followed by incubation with 0.5% Triton X-100 (Sigma-Aldrich, St. Louis, MO,USA) for 10 min at room temperature. The cells were then washed twice and incubated with 1 mL of Click-iT® reaction cocktail for 30 min at room temperature. The cells were then incubated with 100 μL of 5 μg/mL DAPI (Sigma-Aldrich) for an additional 30 min in the dark. After staining, the cells were captured at 600× magnification under a microscope (Olympus, Tokyo, Japan). DAPI is a nuclear stain used to determine total cell counts. Normally, DAPI bound to DNA is most strongly excited by ultraviolet (UV) light at 358 nm and produces the strongest emission in the blue range at 461 nm. Six fields for each sample were randomly captured. EdU-positive cells were counted using ImageJ software (NIH, Bethesda, MD).

### Flow cytometry for apoptosis assay

TM4 cell apoptosis after treatment with different MBPs was analysed using Annexin V-FITC and PI staining kits (Vazyme, Nanjing, China) according to the manufacturer’s requirements. Flow cytometry was performed on a FACSCalibur flow cytometer (BD Biosciences), and the data were analysed using Paint-A-Gate software (Becton-Dickson, San Jose, CA).

### Quantitative PCR (q-PCR) validation analyses of target genes

Analyses of q-PCR were performed as previously described [20]. Total RNA was extracted using TRIzol reagent (Invitrogen, Carlsbad, CA) according to the manufacturer’s protocol. HiScript Q RT SuperMix for q-PCR kit (Vazyme, Nanjing, China) was used for reverse transcription polymerase chain reactions, and then q-PCR assays were conducted with SYBR Green I mix (Takara, Dalian, China) on an ABI ViiA 7 Q-PCR System (Applied Biosystems, Waltham, MA). In all cases, mRNA levels were normalized to the expression of GAPDH, which served as an endogenous control. The relative expression of target genes was calculated by the 2^-△△Ct^ method [22]. The primer sets used in this study are listed in Additional file 1: Table S2.

### Western blotting, coimmunoprecipitation (Co-IP)

Western blotting analyses were executed as previously described [23]. Specific antibody immunological complexes such as Peli2, IRAK1, Bax, Bcl-2, FADD, cl-Caspase 8, cl-Caspase 3, cyclin dependent kinase 1 (CDK1), Caspase 3, p-JNK, JNK, c-Jun, p-c-Jun and GAPDH, were observed by enhanced chemiluminescence. To detect the ubiquitination of IRAK1, an anti-IRAK1 antibody was used to first isolate IRAK1 from TM4 cells by immunoprecipitation, and then ubiquitination of IRAK1 was analysed by immunoblotting using an antibody against ubiquitination (Ub) or K63-Ub.

### Statistical analyses

SPSS 18.0 (SPSS, Chicago, IL) was used for statistical analysis. The normality and homogeneity of variances in the data were checked by using Levene’s test. The Student’s t-test was used for paired comparisons. To compare more than two groups, we used one-way ANOVA with Duncan’s post hoc test. *P* < 0.05 was considered statistically significant.

## Results

### The effect of DBP on the proliferation of SCs

**Fig. 1:**
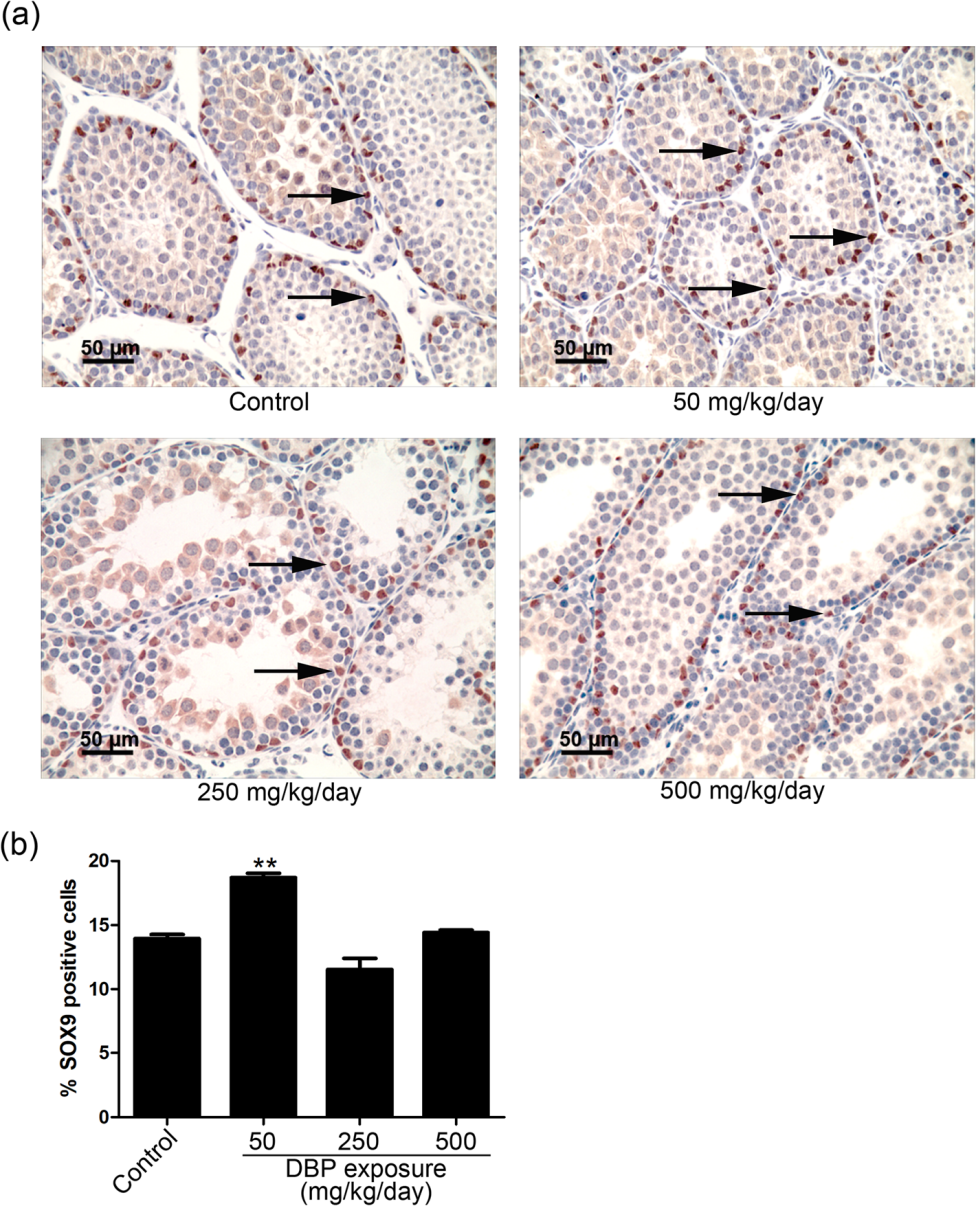
The effect of dibutyl phthalate (DBP) on Sertoli cell (SC) proliferation. **a** The effect of DBP on the number of Sertoli cells per testis in mice after prenatal exposure to DBP. Testicular sections were collected from pups 22 days after mice were exposed in utero (GD12.5 - birth) to corn oil or DBP doses of 50, 250 or 500 mg/kg/day. Immunohistochemical staining for SOX9 was performed (scale bar 50 μm). Arrows represent the expression of SOX9 in the testes of DBP-treated and control male pups. **b** The ratio of SOX9-positive cell was detected by ImageJ (*n* = 6). The results are expressed as the means ± SEM. * *p* < 0.05; ** *p* < 0.01, compared with control

Following in utero exposure to 50 mg/kg/day DBP, the number of SOX9 (a marker of SCs)-positive cells in the testes of pups from the resulting male offspring at postnatal day (PND) 22 was significantly increased compared with the vehicle treatment group; SOX9 was detected by immunohistochemical assay (Fig. 1a, b). These in vivo results suggested that DBP stimulated the proliferation of SCs at a dose of 50 mg/kg/day.

### The effect of MBP on TM4 cell growth and DNA synthesis

**Fig. 2:**
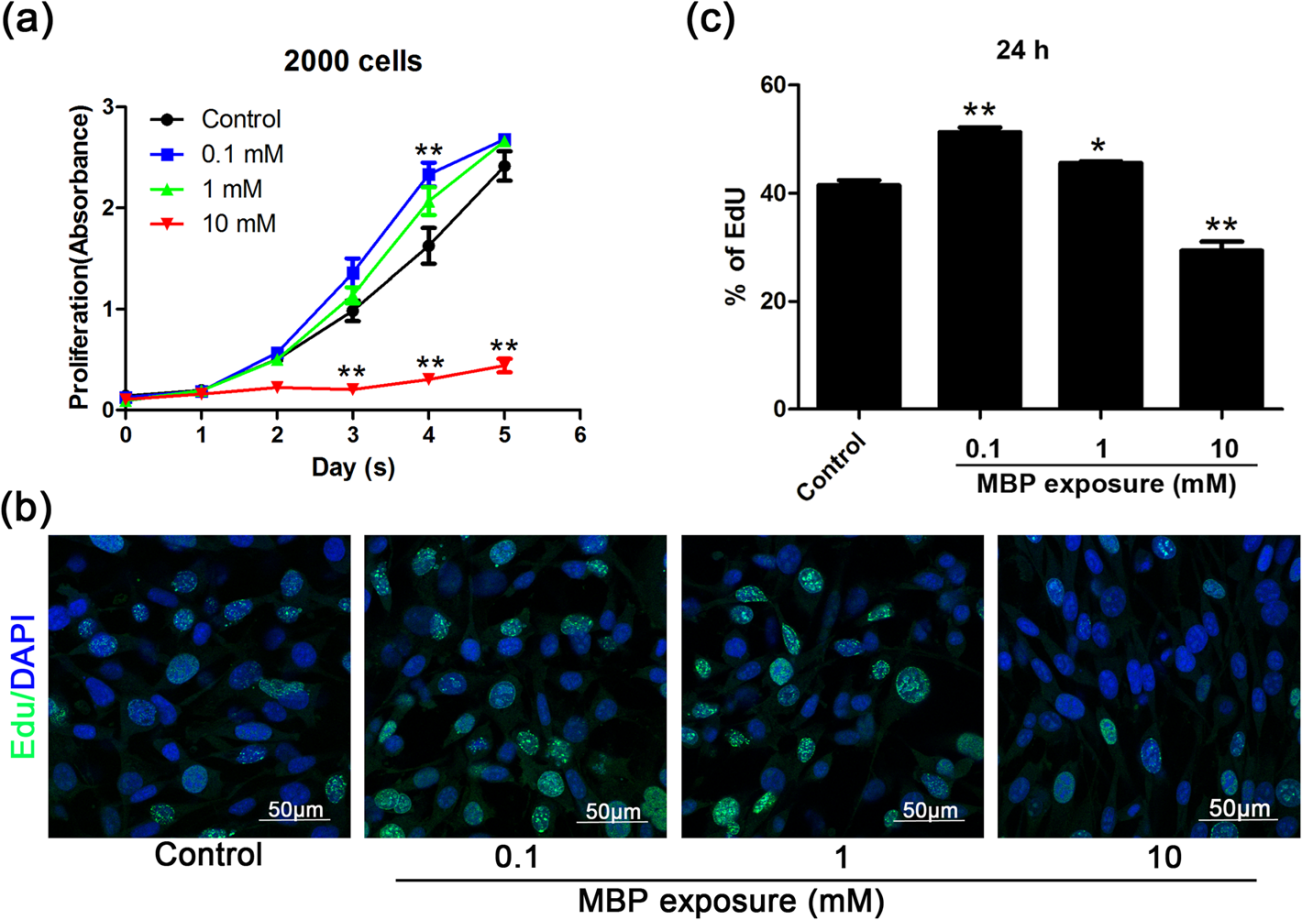
The effect of monobutyl phthalate (MBP) on the proliferation of TM4 cells. **a** Cell viability was measured by CCK-8 assay and showed the viability of TM4 cells after treatment with different concentrations of MBP for 5 days (*n* = 3). **b** Immunofluorescence staining showed EdU incorporation in TM4 cells without treatment (control) or following treatment with 0.1, 1, or 10 mM for 24 h. DAPI was used to counterstain cell nuclei. **c** Quantification of the average percentage of EdU+ cells for B (*n* = 6). The results are expressed as the means ± SEM. * *p* < 0.05; ** *p* < 0.01, compared with control

The results showed that 0.1 mM MBP promoted cell proliferation, but 10 mM MBP inhibited the proliferation of TM4 cells (Fig. 2a). Compared with no treatment, 0.1 mM MBP increased the number of EdU-positive cells, indicating that 0.1 mM MBP promoted DNA synthesis in TM4 cells (Fig. 2b, c). Collectively, these in vitro data confirmed that 0.1 mM MBP stimulated the proliferation of TM4 cells.

### The effect of MBP on the apoptosis of TM4 cells

**Fig. 3:**
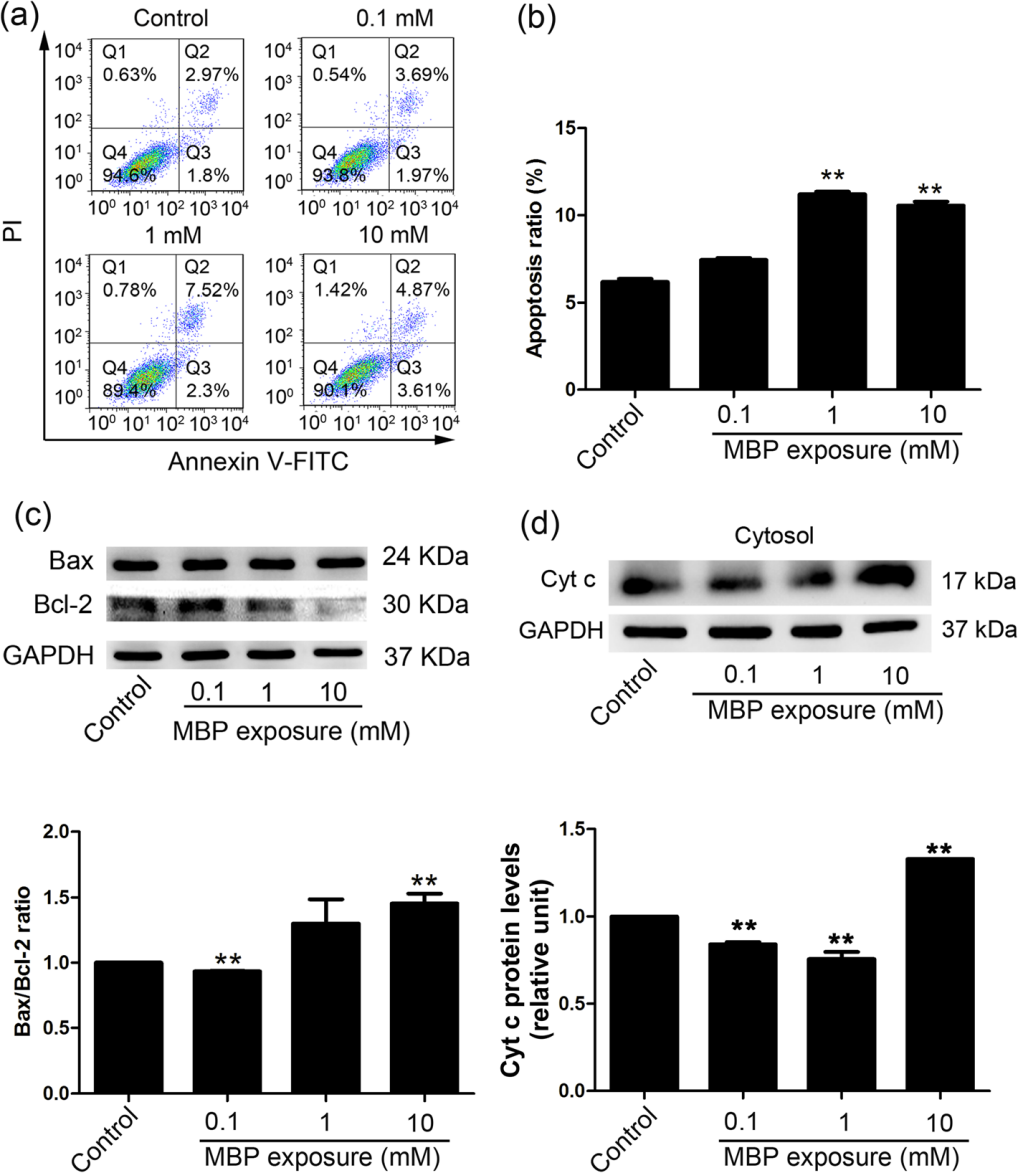
The intrinsic apoptotic pathway participated in MBP-induced apoptosis of TM4 cells. **a** Annexin V-FITC/PI was used to stain apoptotic cells, which were analysed by flow cytometry at 24 h. **b** The level of apoptosis in TM4 cells was calculated (*n* = 3). **c** The protein levels of Bax and Bcl-2 in TM4 cells treated with different concentrations of MBP were measured by Western blotting; the Bax/Bcl-2 ratio was determined by ImageJ (lower panels, *n* = 3). GAPDH was assessed as an internal control. **d** Cytochrome c (Cyt c) release was detected in the cytosolic (Cytosol) fraction of MBP treated TM4 cells 24 h by Western blotting. The densitometry data were quantified with ImageJ (lower panels, *n* = 3). GAPDH was assessed as an internal control. The results are expressed as the means ± SEM. ** *p* < 0.01; * *p* < 0.05

The results of flow cytometry showed that the apoptosis rates of TM4 cells were significantly increased in the 1 mM and 10 mM MBP treatment groups (Fig. 3a, b). To elucidate the mechanism by which MBP induced apoptosis, we examined the effects of MBP on Bcl-2 and Bax expression as well as cytochrome c (Cyt c) release, which are indicators of the intrinsic apoptotic pathways. The Bax/Bcl-2 ratio, as an apoptotic index, is used to evaluate the balance between apoptotic and anti-apoptotic proteins. The results showed that the Bax/Bcl-2 ratio was markedly decreased after exposure to 0.1 mM MBP (Fig. 3c). However, the Bax/Bcl-2 ratio increased in the 10 mM MBP group. Furthermore, the release of Cyt c into the cytosol was significantly increased in TM4 cells after exposure to 10 mM MBP (Fig. 3d, Additional file 1: Fig. S1). We also detected the activation of the extrinsic apoptotic pathway in TM4 cells and found that the extrinsic apoptosis pathway was inhibited after exposure to 10 mM MBP (Additional file 1: Fig. S2). These data indicated that exposure to 10 mM MBP induced apoptosis of TM4 cells by activating the intrinsic apoptotic pathway.

### The effect of DBP/MBP on Peli2 expression

**Fig. 4:**
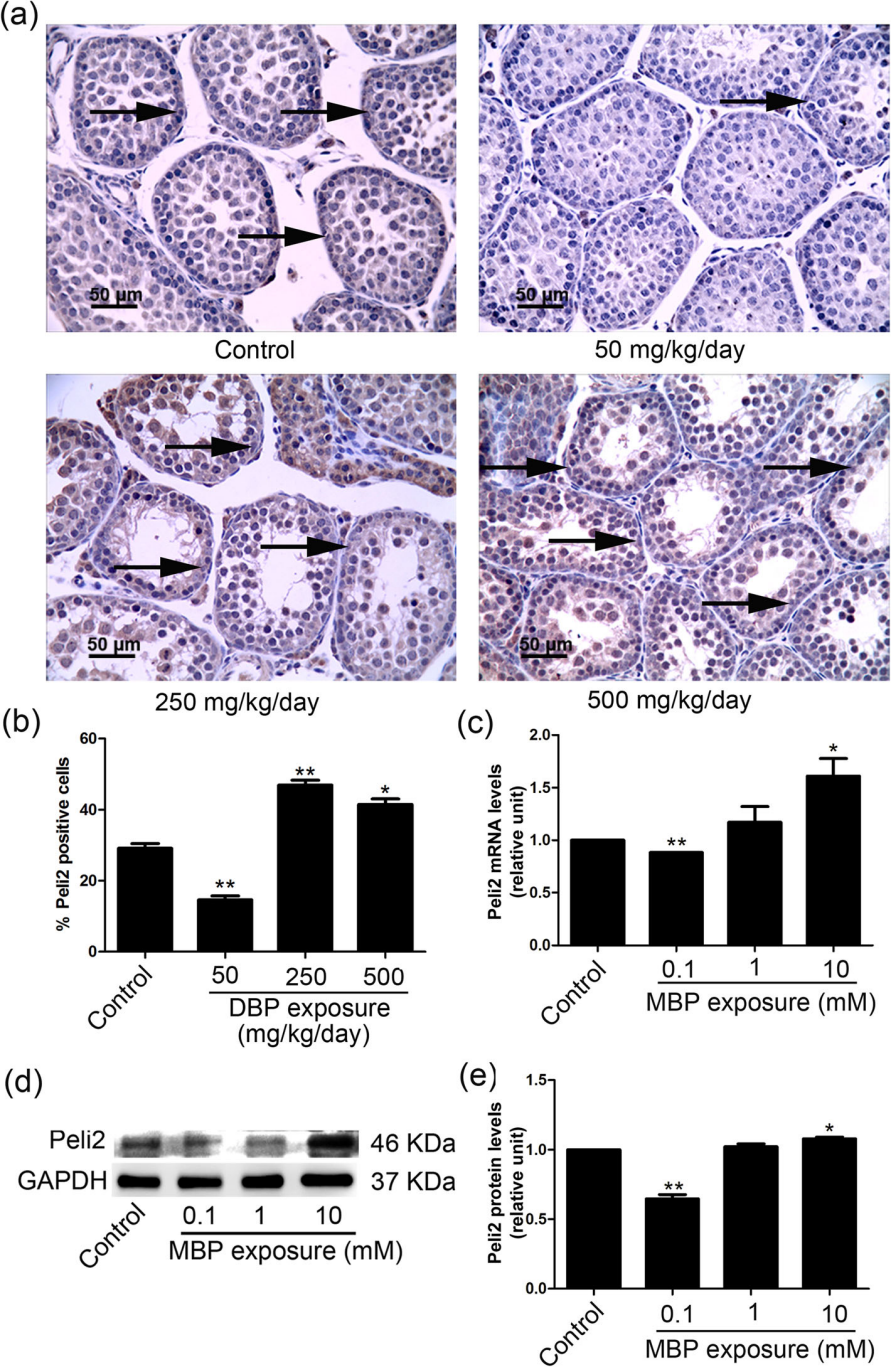
The effects of DBP/MBP exposure on Peli2 expression. **a**, **b** Testicular sections were collected from pups 22 days after they were exposed in utero (GD12.5 - birth) to corn oil or DBP doses of 50, 250 or 500 mg/kg/day. The expression of Peli2 in mouse testicular tissues was carried out by immunohistochemistry. Arrows represent the expression of Peli2 in the testes of DBP-treated and control male pups. The ratio of positive cells was detected by ImageJ (*n* = 6). The expression of Peli2 in SCs after exposure to different concentrations of MBP for 24 h. **c** The mRNA levels of Peli2 were measured with quantitative PCR (q-PCR), and GAPDH was measured as a loading control. **d**, **e** Peli2 protein levels were measured by Western blotting. The densitometry data were quantified with ImageJ (*n* = 3). GAPDH was assessed as an internal control. The results are expressed as the means ± SEM. ** *p* < 0.01; * *p* < 0.05

Based on microarray data in the GEO (Gene Expression Omnibus) database from our previous report [9], Peli2 was chosen for further study because of its important role in cell proliferation. We demonstrated that prenatal exposure to DBP (50 mg/kg/day) reduced the levels of Peli2 in the mouse testes, as shown by immunohistochemical staining (Fig. 4a, b). Moreover, the q-PCR results showed that Peli2 expression in the 0.1 mM MBP group was significantly lower than it was in the control, whereas it was increased in the 10 mM group (Fig. 4c), which was further confirmed by Western blotting (Fig. 4d, e).

### The effect of MBP on the ubiquitination of IRAK1 in TM4 cells

**Fig. 5:**
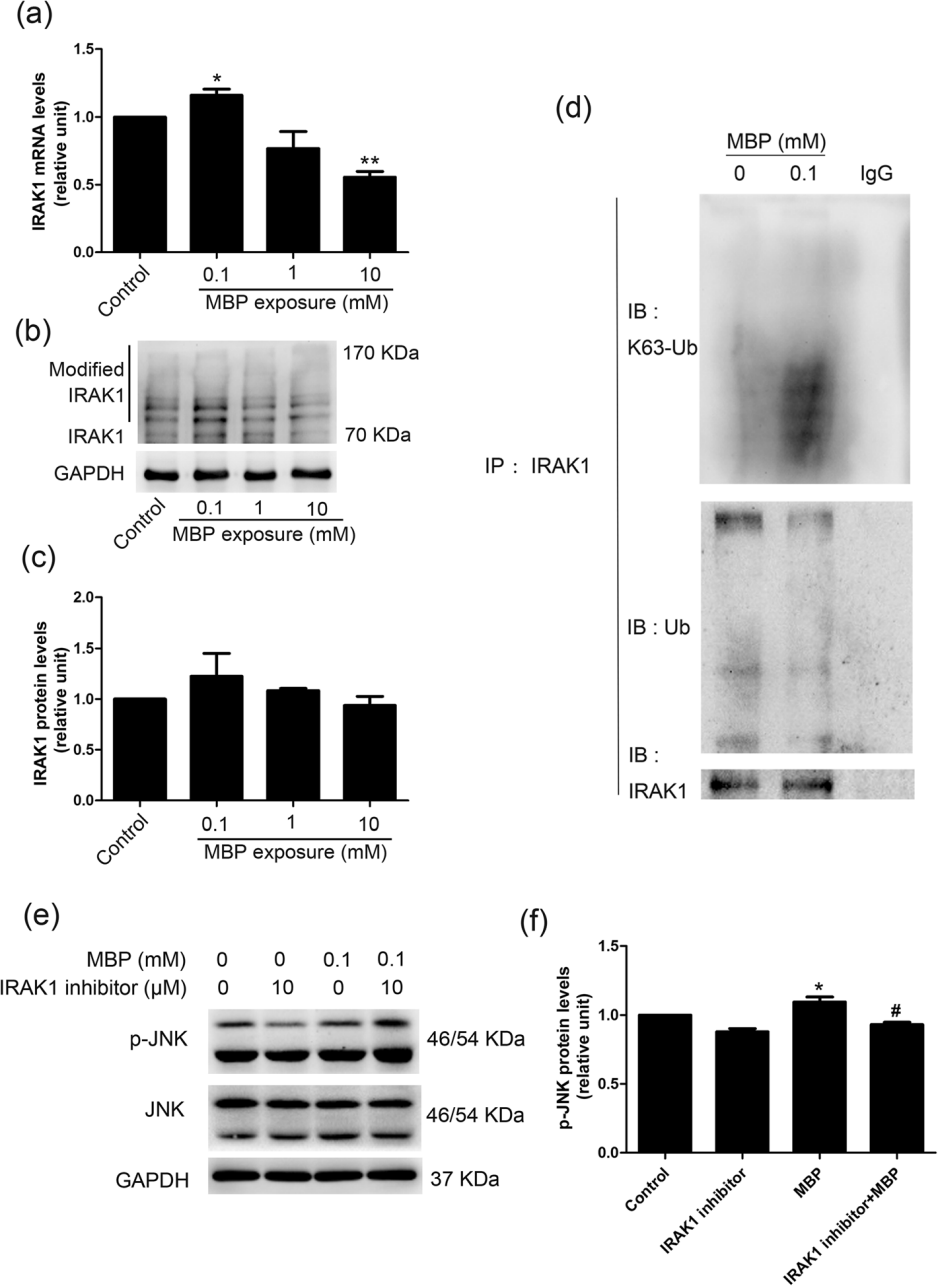
The ubiquitination of IRAK1 in TM4 cells after exposure to MBP. (a-c) The expression of IRAK1 in TM4 cells after exposure to different concentrations of MBP for 24 h. **a** The mRNA levels of IRAK1 were measured with q-PCR, and GAPDH was measured as a loading control. **b**, **c** The protein levels of IRAK1 were measured by Western blotting. The densitometry data were quantified with ImageJ (*n* = 3). GAPDH was assessed as an internal control. **d** MBP (0.1 mM) attenuates IRAK1 ubiquitination and stimulates K63-mediated IRAK1 polyubiquitination. Cell lysates were immunoprecipitated (IP) with anti-IRAK1, which was followed by Western blotting analysis with anti-K63 ubiquitin (K63-Ub), anti-ubiquitin (Ub), and anti-IRAK1 antibodies. **e**, **f** TM4 cells were pretreated with an IRAK1 inhibitor for 1 h, which was followed by 24 h treatment with 0.1 mM MBP. The expression levels of JNK and p-JNK were determined by Western blotting. The densitometry data were quantified with ImageJ (*n* = 3). GAPDH was assessed as an internal control. The results are expressed as the means ± SEM. ** *p* < 0.01; * *p* < 0.05. # *p* < 0.05, vs MBP exposure

Peli2 protein, a RING E3-ubiquitin ligase, can lead to the degradation of IRAK1 by promoting IRAK1 ubiquitination [24, 25], which eventually inhibits the activation of the downstream MAPK/JNK signalling pathway [26]. In this study, we found that the mRNA level of IRAK1 was significantly increased at 0.1 mM MBP, whereas it was suppressed at 10 mM (Fig. 5a). Western blotting results also showed that the protein level of IRAK1 was increased after exposure to 0.1 mM MBP (Fig. 5b, c). A previous study showed that Peli2 played a key role in IL-1- and LPS-induced K63- and K48-linked IRAK1 ubiquitination [27]; thus, we explored IRAK1 ubiquitination by Co-IP. The results showed that after exposure to 0.1 mM MBP, total polyubiquitination of IRAK1 was attenuated compared with that in control cells, while K63-mediated polyubiquitination of IRAK1 was increased (Fig. 5d). To determine whether IRAK1 was upstream of MAPK/JNK, we examined the effects of an IRAK1 inhibitor on MAPK/JNK activation. The IRAK1 inhibitor reduced p-JNK expression at the protein level (Fig. 5e, f). These data suggested that K63-mediated polyubiquitination of IRAK1 might play a key role in DBP/MBP-mediated proliferation of TM4 cells.

### MBP promoted TM4 cell proliferation by MAPK/JNK signalling

**Fig. 6:**
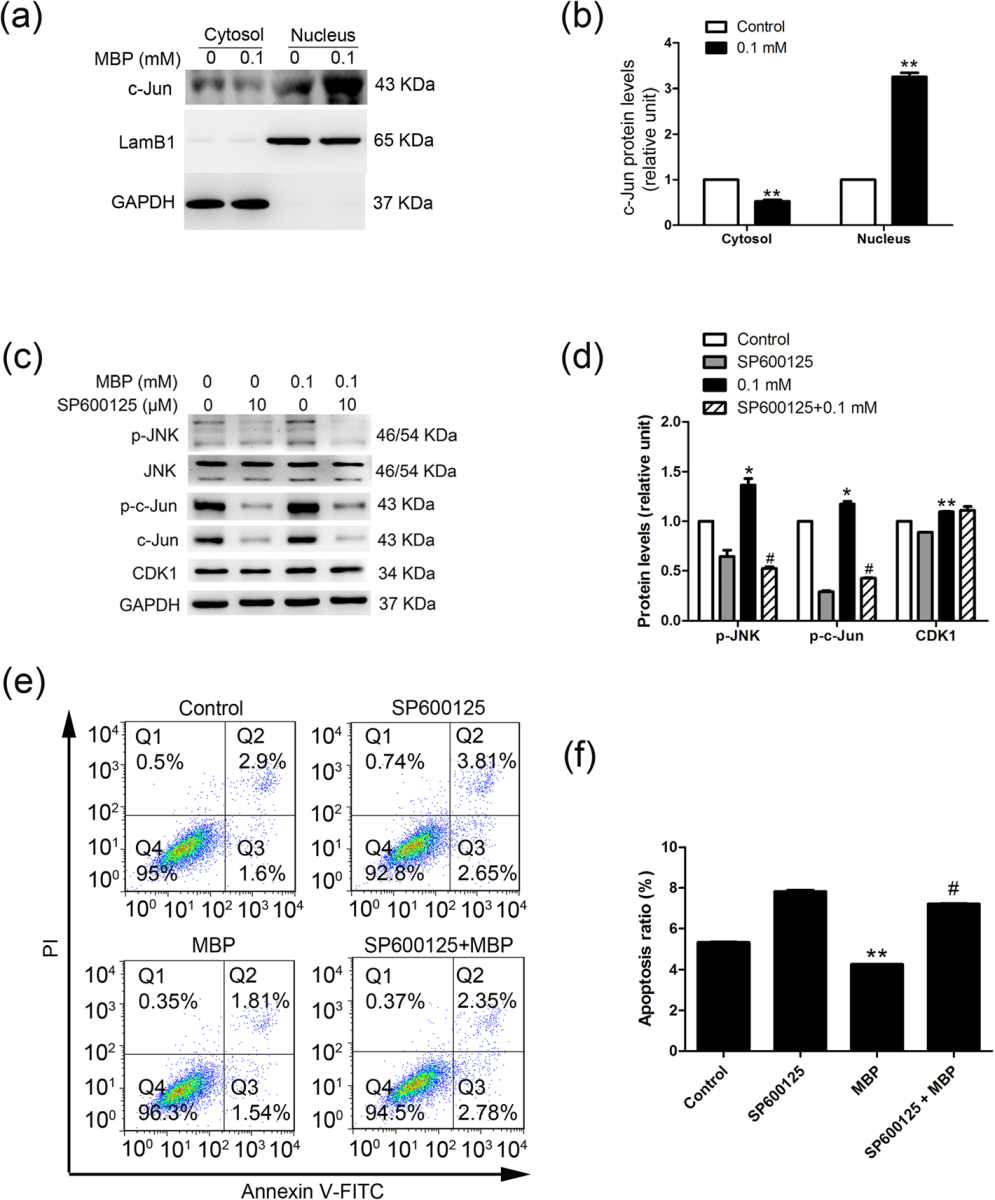
MAPK/JNK signalling is responsible for the proliferation of TM4 cells stimulated by 0.1 mM MBP. **a**, **b** Nuclear and cytosolic fractions were prepared from control and MBP-induced TM4 cells. Levels of c-Jun were analysed by Western blotting. GAPDH and Lamin B1 served as cytosolic and nuclear markers, respectively. The expression levels of c-Jun in the nucleus and cytosol were quantified with ImageJ (right panel; *n* = 3). **c**, **d** TM4 cells were pretreated with SP600125 for 1 h, which was followed by 24 h of treatment with 0.1 mM MBP. (c) Expression levels of c-Jun, p-c-Jun, JNK, p-JNK and cyclin dependent kinase 1 (CDK1) were determined by Western blotting. (d) The densitometry data were quantified with ImageJ (*n* = 3). GAPDH was run as an internal control. **e**, **f** Apoptotic cells were determined by flow cytometry. The level of apoptosis in TM4 cells was calculated (*n* = 3). The data are expressed as the means ± SEM. ** *p* < 0.01; * *p* < 0.05, vs control. # *p* < 0.05, vs MBP exposure

We detected the activation of the MAPK/JNK signalling pathway by assessing downstream members of the pathway by Western blotting. The results showed that the phosphorylation of both JNK (p-JNK) and c-Jun (p-c-Jun) was significantly increased in TM4 cells treated with 0.1 mM MBP (Additional file 1: Fig. S3a, S3b). Additionally, the phosphorylation of c-Jun in the testis after in utero exposure to 50 mg/kg/day DBP was significantly increased (Additional file 1: Fig. S3c, S3d). Furthermore, 0.1 mM MBP also induced marked enrichment of c-Jun in the nuclei of TM4 cells (Fig. 6a, b). To identify whether MAPK/JNK was responsible for the proliferation of TM4 cells, we examined the effects of the JNK inhibitor SP600125 on cell proliferation. The activation of the MAPK/JNK pathway was inhibited after pretreatment with the JNK inhibitor SP600125 (Fig. 6c, d). Furthermore, the MBP-induced increased expression of CDK1 was reduced after pretreatment with the JNK inhibitor SP600125, suggesting that MAPK/JNK participated in MBP-induced TM4 cell proliferation. These results were further confirmed by flow cytometry (Fig. 6e, f).

## Discussion

As an endocrine disruptor found in the environment, DBP is of concern because it is currently widely used in many products, including latex adhesives, cellulose acetate plastics, dyes, personal care products, and coatings for certain oral medications [28]. Humans are exposed to DBP on a daily basis, and daily DBP intake for the general population is 0.007–0.01 mg/kg/day [1]. Detection of the urinary levels of MBP reveal that the metabolites of DBP in women of childbearing age, who are estimated to be exposed to DBP at rates that are over 200 times greater than that of a reference population, as they frequently use oral medications with DBP-incorporated enteric coats [29]. In addition, in some severe cases, DBP metabolites are often found to be nearly 600 times higher than they are in the normal population (10,025 μg/g creatinine vs 17 μg/g creatinine); these patients often require enteric-coated drugs or blood transfusions [30–33]. Furthermore, during the developmental window of foetal mice, the reproductive toxicity of the highest dose of DBP exposure in other studies was mostly 500 mg/kg/day [19, 34, 35]. Therefore, we established 500 mg/kg/day as the highest dose in the in vivo experiments. A previous study showed that 8 mM MBP could inhibit HCG-induced testosterone and insulin-like peptide 3 secretion in cultured testicular interstitial cells in vitro [36]. It was concluded that the in vitro cultured cells are probably insensitive to MBP [36]. Moreover, based on the data regarding the effect of MBP on cell viability in our previous study, the EC_50_ of MBP was determined to be 16.21 mM [21]. Therefore, in this study, the highest concentration of MBP in vitro was set at 10 mM.

The pharmacokinetics of DBP have been investigated in rats [37]. DBP levels in fecal excretion was found to be low, and more than 90% of the dose was excreted via metabolites in the urine within 48 h following either intravenous or oral administration [37, 38]. Most DBP is metabolized to MBP by intestinal hydrolases in the small intestine, and then almost all MBP enters the bloodstream [37, 39]. DBP can directly penetrate the blood-testis barrier [40]. Clewell and his colleagues found that peak MBP concentrations in foetal testes were 72 and 152 μM in the 100 and 500 mg/kg/day DBP exposure groups, respectively [41].

In the present study, we confirmed that prenatal exposure to 50 mg/kg/day DBP promoted SC proliferation. To investigate the mechanism by which DBP/MBP disrupted the growth of immature SCs, we employed TM4 cells derived from immature mouse SCs in an in vitro study. Mouse TM4 cells share many characteristics of SCs and have been widely used as a substitute for primary SCs [42]. Consistent with the in vivo results, 0.1 mM MBP promoted proliferation and DNA synthesis in the TM4 cells, while apoptosis was significantly increased after exposure to 10 mM MBP. We then aimed to investigate the molecular mechanism associated with the proliferation and apoptosis of SCs in MBP-treated SCs at different doses.

Apoptosis is an evolutionarily conserved mechanism for programming cell death, and it occurs in response to some physiological stimuli, cell damage or stress and is an important part of various developmental processes in metazoans [43, 44]. Previously, many investigations found that DBP exposure caused toxicity in several cell types, such as nerve cells, osteoblasts, and germ cells [45–47]. It has been confirmed that MBP exposure causes apoptosis of SCs, but the specific mechanism has not yet been demonstrated [8]. In this study, we analysed the protein levels of key components of the intrinsic pathways (Bax, Bcl-2, and Cyt c) and extrinsic pathways (FADD, caspase 8, and caspase 3) [18]. The results showed that 10 mM MBP could activate the intrinsic pathway, whereas the extrinsic pathway was inhibited. Interestingly, the expression of FADD was increased after exposure to 0.1 mM MBP (Additional file 1: Fig. S2a). A previous study showed that FADD played a role in regulating most of the signalosome complexes, causing it to emerge as a newly identified actor in innate immunity, inflammation, and cancer development [48]. Therefore, we speculated that FADD might be involved in other physiological processes after exposure to MBP at a concentration of 0.1 mM.

Pellino proteins have various regulatory roles in cell growth, for example, murine genetic models have revealed roles for Peli1 in lung carcinogenesis [49] and for Peli3 in TNF-induced cell killing [50]. However, there is a notable lack of insight into the physiological roles of Peli2. It was illustrated that polyubiquitination of both IL-1/LPS-induced K63- and K48-linked IRAK1 was decreased in Peli2-knockdown cells [27]. In our study, we found that, with decreasing Peli2 expression, the total ubiquitination level was reduced, while K63-linked IRAK1 polyubiquitination was increased after exposure to 0.1 mM MBP. Therefore, we hypothesized that the decreasing IRAK1 ubiquitination was mainly due to K48-ubiquitination, which resulted in the degradation of IRAK1. Studies on Peli2 revealed a role for Peli2 in IL-1/LPS-induced activation of the MAPK/JNK pathway [27, 51]. Our data also found that 0.1 mM MBP activated IRAK1 and the downstream MAPK/JNK signalling pathway, suggesting that 0.1 mM MBP could promote immature SC growth through the Peli2/IRAK1/MAPK/JNK pathway. Taken together, it was concluded that 0.1 mM MBP promoted the abnormal proliferation of SCs by inhibiting the expression of Peli2, disrupting the balance of IRAK1 ubiquitination, and activating the downstream MAPK/JNK signalling pathway.

## Conclusions

**Fig. 7:**
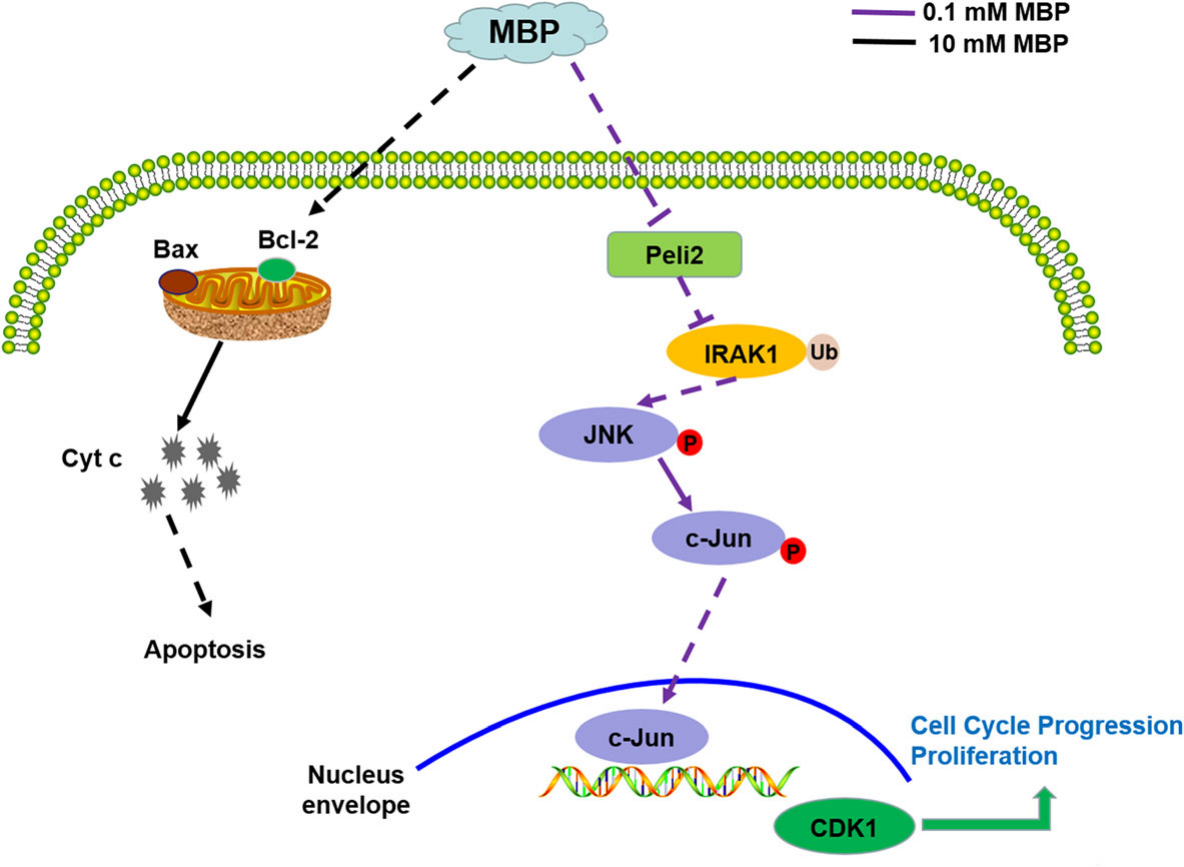
Proposed model for MBP-induced abnormal cell growth of juvenile SCs. MBP at 0.1 mM inhibits the expression of Peli2, leading to K63 ubiquitination of IRAK1, which activates the MAPK/JNK signalling pathway and promotes SC proliferation. MBP at 10 mM led to SC apoptosis through intrinsic apoptotic signalling pathways

In summary, we first confirmed that DBP/MBP stimulated the proliferation of SCs in vitro and in vivo at a relatively low concentration range. Then, we found that downregulated Peli2 resulted in increased K63 ubiquitination of IRAK1, which activated MAPK/JNK signalling pathways in TM4 cells treated with 0.1 mM MBP. In addition, we showed that 10 mM MBP caused apoptosis of TM4 cells by activating the intrinsic apoptotic pathway. A descriptive outline of this study is shown in Fig. 7.

## Supplementary information

**Additional file 1 Table S1.** Specifications of primary antibodies. **Table S2.** Primers used for q-PCR. **Figure S1.** Cytochrome C (Cyt C) released was induced by MBP at 10 mM group. **Figure S2.** The extrinsic apoptotic pathway do not participated in MBP-induced apoptosis of TM4 cells. **Figure S3.** MBP induces the activation of MAPK/JNK-associated protein in TM4 cells.

## Data Availability

All data generated during this study are included in this published article and its additional files.

## Abbreviations

DBP: Dibutyl phthalate
MBP: Monobutyl phthalate
SCs: Sertoli cells
Peli2: Pellino 2
IRAK1: Interleukin 1 receptor-associated kinase 1
