# Supplementary material for: Dibutyl phthalate promotes juvenile Sertoli cell proliferation by decreasing the levels of the E3 ubiquitin ligase Pellino 2
Source: Environ Health. 2020 Aug 1;19:87. doi: 10.1186/s12940-020-00639-1 (PMC7395429; doi:10.1186/s12940-020-00639-1)
Supplement: Supplementary file 1 — Additional file 1 Table S1. Specifications of primary antibodies. Table S2. Primers used for q-PCR. Figure S1. Cytochrome C (Cyt C) released was induced by MBP at 10 mM group. Figure S2. The extrinsic apoptotic pathway do not participated in MBP-induced apoptosis of TM4 cells. Figure S3. MBP induces the activation of MAPK/JNK-associated protein in TM4 cells. [file 12940_2020_639_MOESM1_ESM.docx]

**Additional file**

**Dibutyl phthalate promotes juvenile Sertoli cell proliferation by decreasing the levels of the E3 ubiquitin ligase Pellino 2**

Tan Ma^1, 2^, Jiwei Hou^1, 2^, Yuan Zhou^1, 2^, Yusheng Chen^1, 2^, Jiayin Qiu^1, 2^, Jiang Wu^1, 2^, Jie Ding^1, 2^, Xiaodong Han^1, 2^, Dongmei Li^1, 2*^

^1^ Immunology and Reproduction Biology Laboratory & State Key Laboratory of Analytical Chemistry for Life Science, Medical School, Nanjing University, Nanjing, Jiangsu 210093, China

^2^ Jiangsu Key Laboratory of Molecular Medicine, Nanjing University, Nanjing, Jiangsu 210093, China

**Corresponding Author at:** Immunology and Reproduction Biology Laboratory & State Key Laboratory of Analytical Chemistry for Life Science, Medical School, Nanjing University, Nanjing, Jiangsu 210093, China

***E-mail addresses*:** [lidm@nju.edu.cn](mailto:lidm@nju.edu.cn) **(**Dongmei Li)

**Table S1.** Specifications of primary antibodies

| **Antibody** | **Species** | **Company** | **Catalog** | **Dilution** |
| --- | --- | --- | --- | --- |
| Anti-Peli2 | Rabbit Polyclonal Ab | Proteintech | 16097-1-AP | 1:1000 (WB)  1:50 (IF)  1:50 (IHC) |
| Anti-IRAK1 | Mouse mAb | Santa Cruz | sc-5288 | 1:1000 (WB) |
| Anti-Bax | Rabbit Polyclonal Ab | Proteintech | 50599-2-Ig | 1:2000 (WB) |
| Anti-Bcl-2 | Rabbit Polyclonal Ab | Proteintech | 12789-1-AP | 1:2000 (WB) |
| Anti-Cyt c | Mouse mAb | Proteintech | 66264-1-Ig | 1:5000 (WB)  1:100 (IF) |
| Anti-FADD | Mouse mAb | Santa Cruz | sc-271748 | 1:1000 (WB) |
| Anti-cl-Caspase 8 | Rabbit mAb | CST | #8592 | 1:1000 (WB) |
| Anti-cl-Caspase 3 | Rabbit Polyclonal Ab | Proteintech | 25546-1-AP | 1:1000 (WB) |
| Anti-Caspase 3 | Rabbit Polyclonal Ab | Proteintech | 19677-1-AP | 1:1000 (WB) |
| Anti-CDK1 | Rabbit Polyclonal Ab | Proteintech | 19532-1-AP | 1:2000 (WB) |
| Anti-JNK | Mouse mAb | Santa Cruz | sc-7345 | 1:200 (WB) |
| Anti-p-JNK | Rabbit Polyclonal Ab | Boster | BM4380 | 1:200 (WB) |
| Anti-c-Jun | Rabbit Polyclonal Ab | Absci | #AB21003 | 1:1000 (WB) |
| Anti-p-c-Jun | Rabbit Polyclonal Ab | Absci | #AB11003 | 1:1000 (WB)  1:50 (IHC) |
| Anti-SOX9 | Rabbit mAb | abcam | Ab185230 | 1:2000 (IHC) |
| Anti-GAPDH | Mouse mAb | Absci | #AB40493 | 1:3000 (WB) |

**Table S2.** Primers used for real-time PCR

| **Gene** | **Primer (forward/reverse)** |
| --- | --- |
| Peli2 | F: AGAAAGCCCTATCGACTTCGT  R: ATGGTGCTCTGTGTGATCTGG |
| IRAK1 | F: AGCCGAGGTCTGCATTACATT  R: TGGCAGTCTGGATAACTGATGA |
| GAPDH | F: AGGTCGGTGTGAACGGATTTG  R: TGTAGACCATGTAGTTGAGGTCA |

**
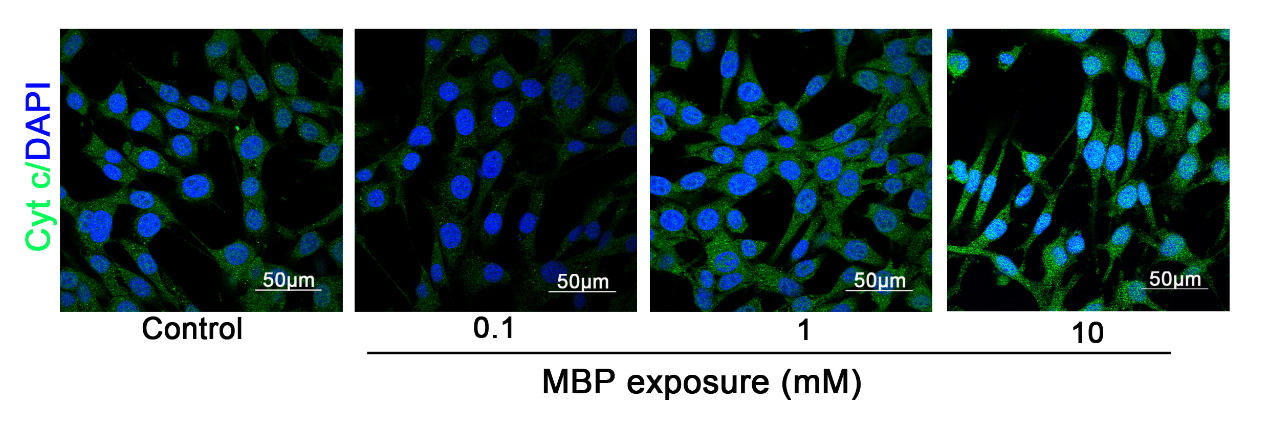
**

**Fig. S1** Cytochrome C (Cyt C) released was induced by MBP at 10 mM group. The release of Cyt c was measured by immunofluorescence. Cyt c was revealed with secondary 488-labeled antibody; nuclei was revealed by DAPI staining.

**
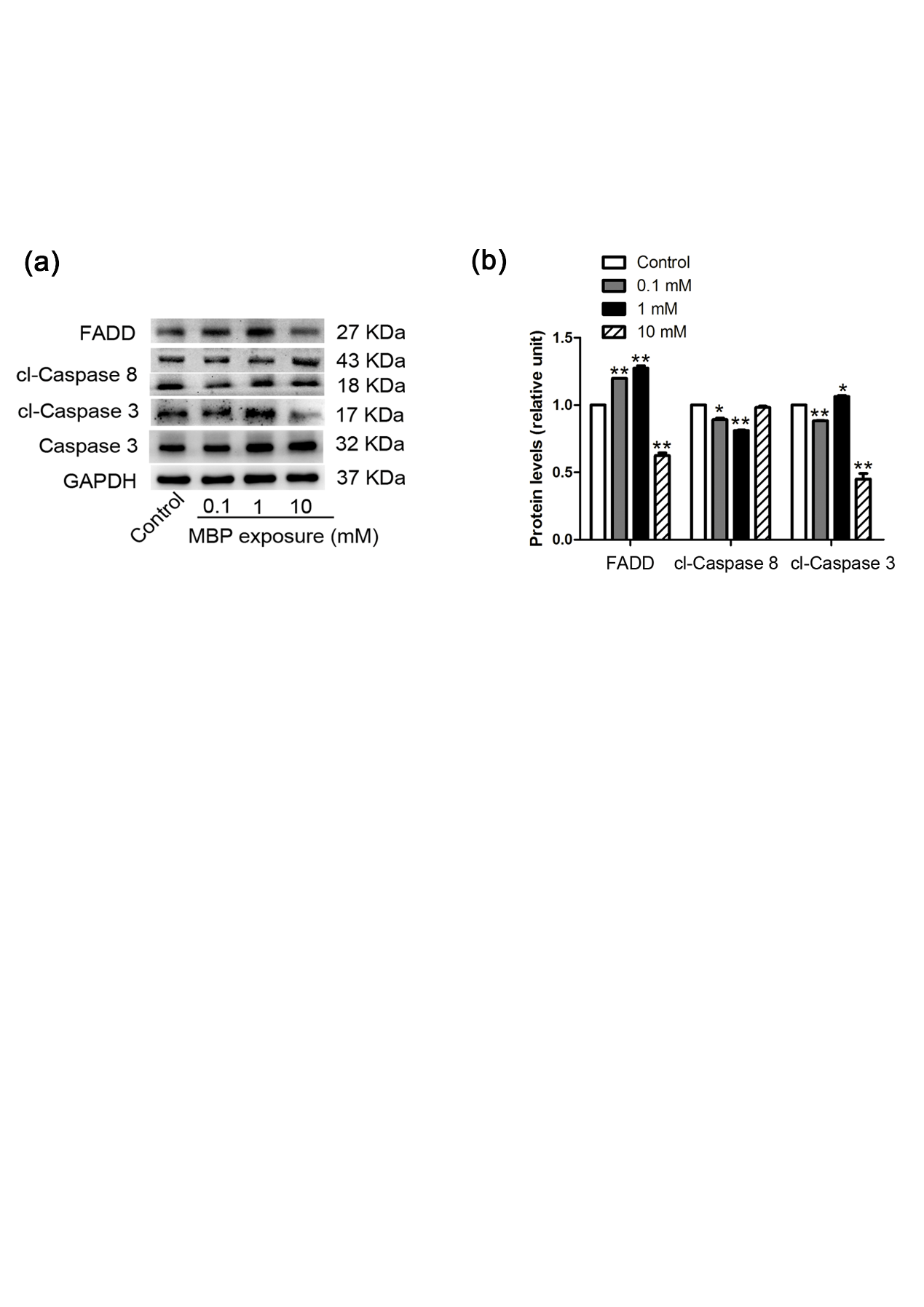
**

**Fig. S2** The extrinsic apoptotic pathway do not participated in MBP-induced apoptosis of TM4 cells. (a) Expression levels of FADD, cl-Caspase 8, cl-Caspase 3 and Caspase 3 were determined by Western blotting. (b) The expression levels were quantified with ImageJ (n = 3). GAPDH was run as an internal control. The results are expressed as the means ± SEM. ** *p* < 0.01; * *p* < 0.05.

**
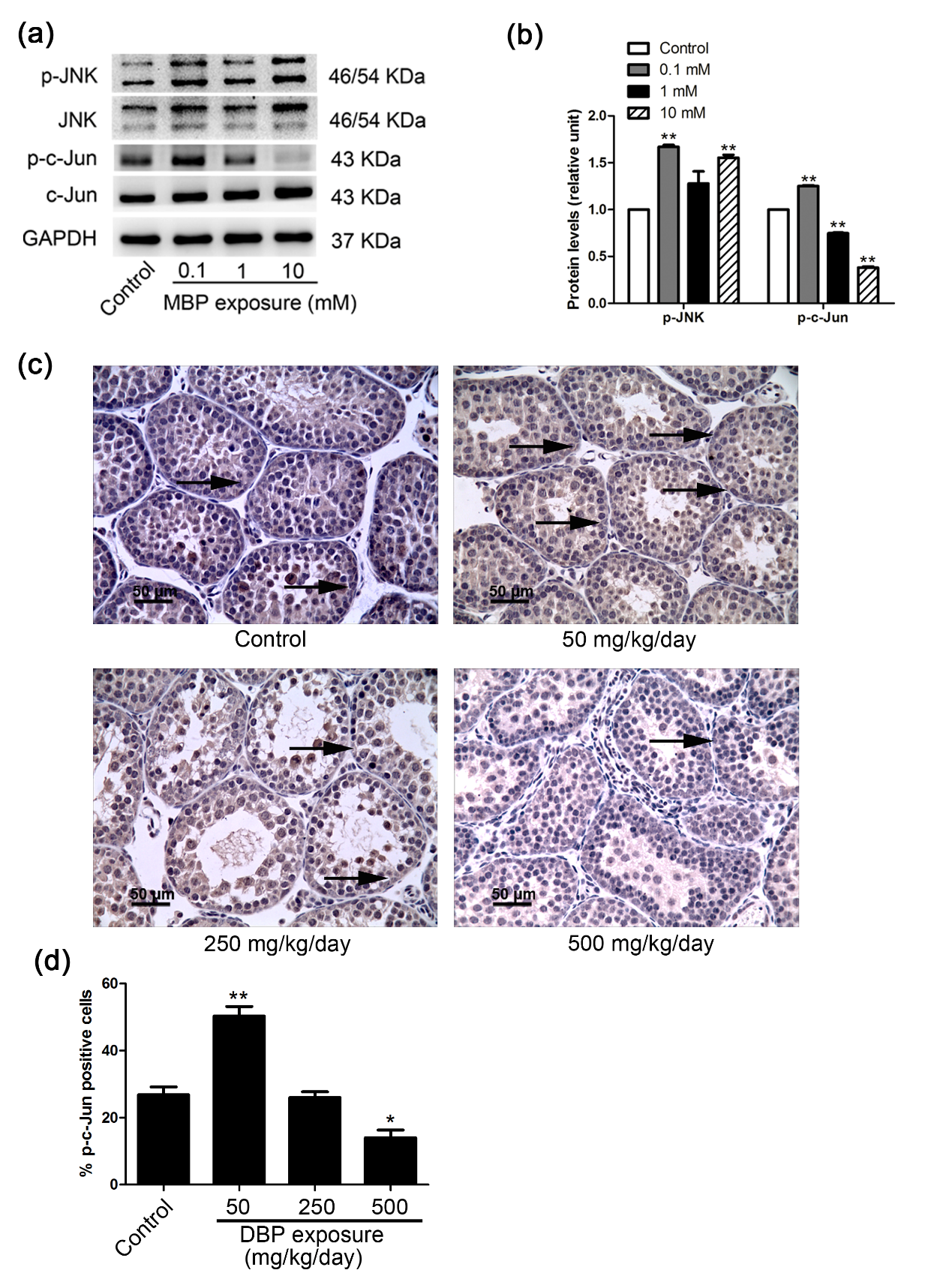
**

**Fig. S3** MBP induces the activation of MAPK/JNK-associated protein in TM4 cells. (a) The protein levels of total c-Jun, phosphorylated c-Jun (p-c-Jun), phosphorylated JNK (p-JNK), total JNK in TM4 cells treated with various concentrations of MBP were measured by Western blotting. (b) The expression levels were quantified with ImageJ (n = 3). GAPDH was run as an internal control. (c, d) Testicular sections were collected from pups 22 days after they were exposed *in utero* (GD12.5 - birth) to corn oil or DBP doses of 50, 250 or 500 mg/kg/day. The expression of p-c-Jun in mouse testicular tissues was carried out by immunohistochemistry. Arrows represent the expression of p-c-Jun in the testes of DBP-treated and control male pups. The ratio of positive cell was detected by ImageJ (n = 6). The results are expressed as the means ± SEM. ** *p* < 0.01; * *p* < 0.05.
